# EARLY CHILDHOOD EXPERIENCES AND LATE LIFE COGNITIVE FUNCTION AMONG OLDER CHINESE ADULTS

**DOI:** 10.1093/geroni/igae098.3837

**Published:** 2024-12-31

**Authors:** Duy Nguyen, Rui Liu, Limei Chen, Yookyong Lee

**Affiliations:** Sacred Heart University, Teaneck, New Jersey, United States; Sacred Heart University, Fairfield, Connecticut, United States; George Mason University, Fairfax, Virginia, United States; University of Alabama - Birmingham, Birmingham, Alabama, United States

## Abstract

Extensive research has documented the negative impacts of adverse childhood experiences on individual well-being, yet Asian older adults have been overlooking. For the current study, we used the China Health and Retirement Longitudinal Study (CHARLS), a national, longitudinal study of representative residents and applied the Life Course Perspective to test how childhood conditions and social relationship indicators affect cognitive health at later life. This study combined data from three datasets: Wave 1 (2011), The Life History Survey Data (2014), and Wave 5 (2020). The study sample (N=9,941) included respondents 50 and over at baseline, without cognitive impairment in the baseline, and still living at Wave 5. Women constituted 52.4% of the sample, and the mean age was 60.2 (SD=6.77). Two outcome variables (self-rated memory and cognitive health status) were used in data analyses. The multivariate analyses results showed that weak neighborhood quality and social cohesion, poor social relationships, poor childhood health status were associated with worse cognitive function. The study findings demonstrate the effects of early childhood conditions on late-life cognitive function. As aging in China accounts for most global aging, public health approaches that stimulate positive childhood memories can have a meaningful protective effect for older adult well-being. Our findings underscore the global importance of childhood health, childhood experiences, and social relationships, which are not only those older adults in China but globally. Further cross-national research is needed to understand the diversity in sociohistorical experiences across global aging populations.

